# *In situ* synthesis, enhanced luminescence and application in dye sensitized solar cells of Y_2_O_3_/Y_2_O_2_S:Eu^3+^ nanocomposites by reduction of Y_2_O_3_:Eu^3+^

**DOI:** 10.1038/srep37133

**Published:** 2016-11-22

**Authors:** Guohai Yuan, Mingxia Li, Mingqi Yu, Chungui Tian, Guofeng Wang, Honggang Fu

**Affiliations:** 1 Key Laboratory of Functional Inorganic Material Chemistry, Ministry of Education, School of Chemistry and Materials Science, Heilongjiang University, Harbin, 150080, China

## Abstract

Y_2_O_3_/Y_2_O_2_S:Eu^3+^ nanocomposites were successfully prepared by reducing Y_2_O_3_:Eu^3+^ nanocrystals. The obtained Y_2_O_3_/Y_2_O_2_S:Eu^3+^ nanocomposites not only can emit enhanced red luminescence excited at 338 nm, but also can be used to improve the efficiency of the dye sensitized solar cells, resulting an efficiency of 8.38%, which is a noticeable enhancement of 12% compared to the cell without Y_2_O_3_/Y_2_O_2_S:Eu^3+^ nanocomposites. The results of the incident photon to current, dynamic light scattering, and diffuse reflectance spectra indicated that the enhancement of the cell efficiency was mainly related to the light scattering effect of Y_2_O_3_/Y_2_O_2_S:Eu^3+^ nanocomposites. As a phosphor powder, the emission at ~615 nm of Y_2_O_3_/Y_2_O_2_S:Eu^3+^ was split into two sub-bands. Compared with Y_2_O_3_:Eu^3+^, the ^5^D_0_ → ^7^F_0_ and ^5^D_0_ → ^7^F_1_ emissions of Y_2_O_3_/Y_2_O_2_S:Eu^3+^ showed a little red-shift.

Rare earth (RE) compounds were extensively applied in the fields of high-performance magnets, luminescence devices, catalysts, and other functional materials. Most of these functions depend strongly on the composition and structure of materials^1^,^2^,^3^,^4^,^5^. In particular, nano-sized luminescent materials have attracted considerable attention since Bhargava *et al*. reported that doped nanocrystalline phosphors yielded high luminescence efficiencies^6^,^7^,^8^,^9^. With rapidly shrinking size, nanomaterials usually exhibit novel physical and chemical properties due to their extremely small size and relatively large specific surface areas^10^,^11^,^12^,^13^.

It is well known that host material is an important factor to obtain high efficient luminescent properties. Among various host materials, Y_2_O_3_ not only has good chemical and photochemical stabilities and high melting points, but also can be easily doped with RE ions. Especially, Y_2_O_3_:Eu^3+^ phosphor is used for high efficiency cathode-ray tubes and field emission displays because of its excellent luminescence efficiency under ultraviolet excitation^14^,^15^,^16^,^17^. Y_2_O_2_S:Eu^3+^ has been used as a red “no mill” phosphor for decades. Its high brightness, excellent color definition, and linear response in the wide range of current density make it promising for the future generation of display equipment^18^,^19^,^20^,^21^,^22^.

Composite materials formed by combining two or more materials could present complementary properties that have shown important technological applications^23^,^24^. However, Y_2_O_3_/Y_2_O_2_S composite nanocrystals have never been reported. It is well known that the excitation spectrum of Y_2_O_3_:Eu^3+^ was dominated by the excitation band centered at 259 nm, while that of Y_2_O_2_S:Eu^3+^ was dominated by the excitation band centered at 338 nm. In addition, the emission spectrum of Y_2_O_3_:Eu^3+^ was dominated by the emission at ~615 nm, while that of Y_2_O_2_S:Eu^3+^ was dominated by the emission at ~630 nm. And thus, novel luminescent properties could be obtained by combining Y_2_O_3_:Eu^3+^ and Y_2_O_2_S:Eu^3+^.

In the past decade, the dye-sensitized solar cell (DSSC) has become one of the most promising solar cells in the renewable energy research and development field for its potentially low fabrication cost and relatively good efficiency^25^,^26^,^27^. The concept of integrating a down-conversion layer into a solar cell has attracted significant attention because it not only can remove the load of spectral matching from the semiconductor itself, minimize the thermalization losses in cells, and move this task to a separation component, but also can offer the opportunity to improve light harvesting and thereby the efficiency of the solar cells^28^,^29^,^30^. Herein, we successfully prepared Y_2_O_3_/Y_2_O_2_S:Eu^3+^ nanocomposites by reducing Y_2_O_3_:Eu^3+^ nanocrystals for the first time. The obtained Y_2_O_3_/Y_2_O_2_S:Eu^3+^ nanocomposites not only can present excellent luminescence performance, but also can be chosen to design TiO_2_-Y_2_O_3_/Y_2_O_2_S:Eu^3+^ composite cell with improved photoelectrochemical properties. The mechanism for the enhancement of the cell efficiency was investigated in detail.

## Discussion

**Figure 1:**
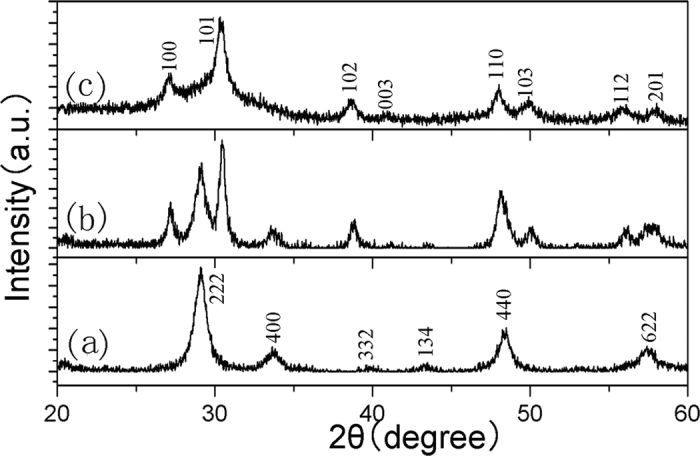
XRD patterns of (**a**) Y_2_O_3_:Eu^3+^, (**b**) Y_2_O_3_/Y_2_O_2_S:Eu^3+^, and (**c**) Y_2_O_2_S:Eu^3+^.

Sample numbers and corresponding experimental conditions are given in Table 1. When the content of sulfur powder was 1.0 or 1.5 g, Y_2_O_3_/Y_2_O_2_S:Eu^3+^ was obtained. When the content of sulfur powder was 2.0 g, Y_2_O_2_S:Eu^3+^ was obtained. It is noted that the diffraction peaks of Y_2_O_3_:Eu^3+^ can be indexed to the cubic phase Y_2_O_3_ (JCPDS 43-1036), and the diffraction peaks of Y_2_O_2_S:Eu^3+^ can be indexed to the hexagonal phase Y_2_O_2_S (JCPDS 24-1424). The corresponding XRD patterns of Y_2_O_3_:Eu^3+^, Y_2_O_3_/Y_2_O_2_S:Eu^3+^, and Y_2_O_3_/Y_2_O_2_S:Eu^3+^ nanocomposites were shown in Fig. 1.

**Figure 2:**
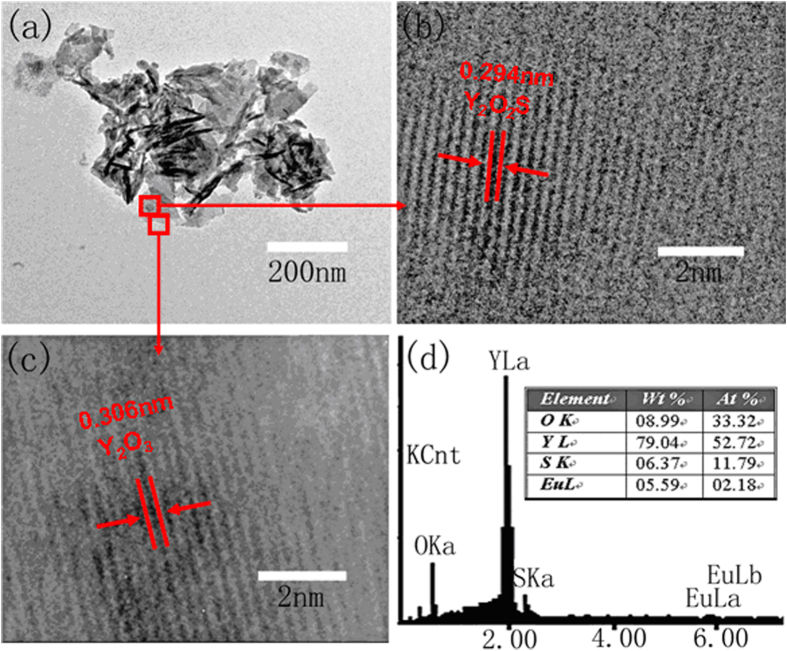
(**a**–**c**) TEM and HRTEM images of Y_2_O_3_/Y_2_O_2_S:Eu^3+^ nanocomposites. (**d**) EDX spectrum of Y_2_O_3_/Y_2_O_2_S:Eu^3+^ nanocomposites.

Figure 2(a–c) shows the TEM and HRTEM images of Y_2_O_3_/Y_2_O_2_S:Eu^3+^ nanocomposites. Typical HRTEM image shows interplanar spacings of 0.306 and 0.294 nm corresponding to the (222) plane of Y_2_O_3_ and (101) plane of Y_2_O_2_S, respectively. The results indicated that Y_2_O_3_:Eu^3+^ and Y_2_O_2_S:Eu^3+^ coexist in the Y_2_O_3_/Y_2_O_2_S:Eu^3+^ nanocomposites. In order to determine the content of Y_2_O_2_S:Eu^3+^ in nanocomposites, Y_2_O_3_/Y_2_O_2_S:Eu^3+^ nanocomposites were measured using energy dispersive X-ray (EDX) analysis, as shown in Fig. 2(d). The result indicated that the content of Y_2_O_2_S:Eu^3+^ was 43 mol% in Y_2_O_3_/Y_2_O_2_S:Eu^3+^ nanocomposites.

**Figure 3:**
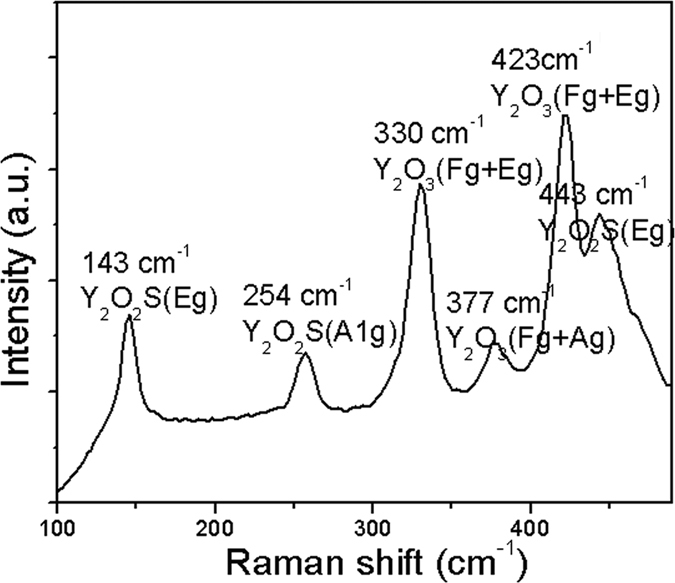
Raman spectrum of Y_2_O_3_/Y_2_O_2_S:Eu^3+^ nanocomposites.

Figure 3 shows the Raman spectrum of Y_2_O_3_/Y_2_O_2_S:Eu^3+^ nanocomposites. The results further indicated that Y_2_O_3_:Eu^3+^ and Y_2_O_2_S:Eu^3+^ coexist in Y_2_O_3_/Y_2_O_2_S:Eu^3+^ nanocomposites. The Raman active modes of Y_2_O_3_:Eu^3+^ are featured by three bands at about 300~430 cm^−1^, which can be assigned to the Fg+Eg and Fg+Ag modes. The Raman active modes of Y_2_O_2_S:Eu^3+^ observed at 143, 254, 443 cm^−1^ were cause by the intense Eg, A1g, and Eg modes, respectively.

**Figure 4:**
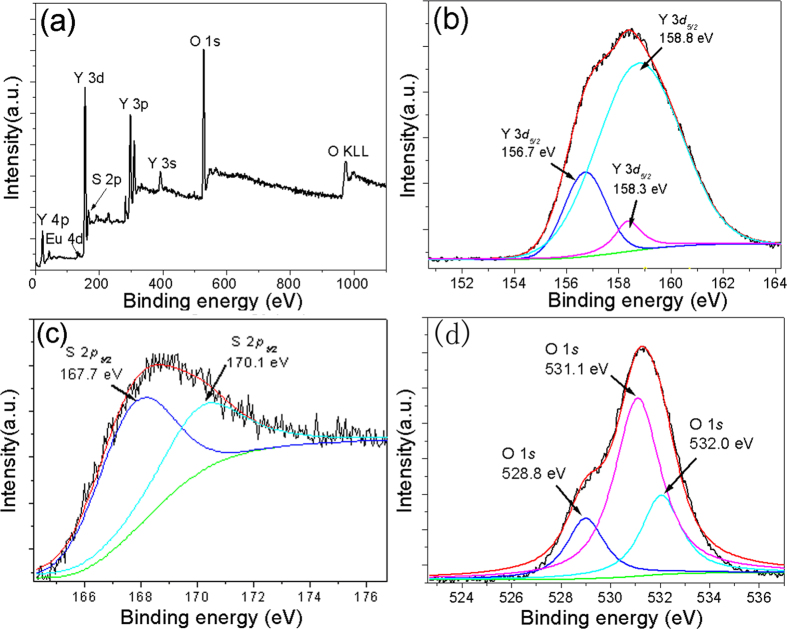
The XPS spectra of Y_2_O_3_/Y_2_O_2_S:Eu^3+^ nanocomposites.

Figure 4 shows the XPS spectrum of Y_2_O_3_/Y_2_O_2_S:Eu^3+^ nanocomposites. Obviously, Y^3+^ was identified by its Y 3 s, Y 3p, Y 3d, and Y 4 P speaks, O^2−^ was identified by the O 1 s and O KLL peaks, Eu^3+^ was identified by the Eu 4d peak, and S^2+^ was identified by the S 2p peak. The Y 3d_3/2_ spectral peaks were at 156.7, 158.3, and 158.8 eV, and the S 2p_2/3_ spectral peaks were at 167.7 and 170.1 eV. In addition, the O 2 s spectrum can be fitted by three peaks located at 628.8, 531.1, and 532.0 eV.

**Figure 5:**
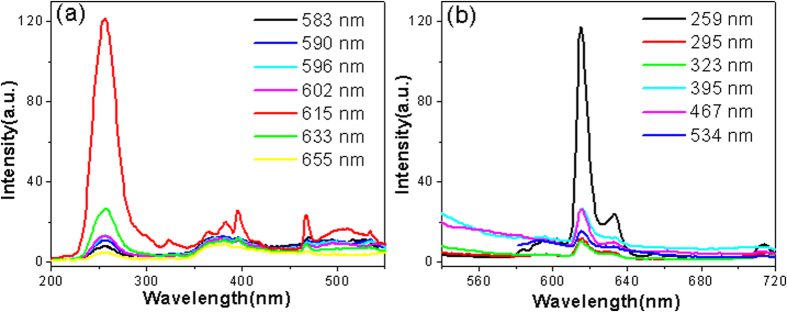
The (**a**) excitation and (**b**) emission spectra of the Y_2_O_3_:Eu^3+^ nanocrystals.

For comparison, the luminescence properties of the Y_2_O_3_:Eu^3+^ (without sulfuration) nanocrystals were investigated first, as shown in Fig. 5. For the excitation spectra of Y_2_O_3_, the broad band extending from 200 to 300 nm is assigned to the charge transfer transition from the 2p orbital of O^2−^ to the 4 f orbital of Eu^3+^, which is related closely to the covalency between O^2−^ and Eu^3+^ and the coordination environment around Eu^3+^. The sharp lines in Fig. 5(a) correspond to the f-f transitions of the Eu^3+^ ions. Figure 5(b) shows the emission spectra of Y_2_O_3_:Eu^3+^ excited at different wavelengths. It is found that the peak at ~615 nm of Y_2_O_3_:Eu^3+^ was much stronger than that at ~630 nm. When the excitation wavelength was 259 nm, the emission intensities were the strongest.

**Figure 6:**
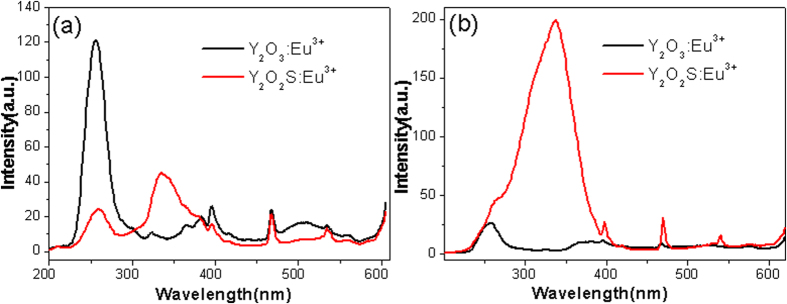
Excitation spectra of Y_2_O_3_:Eu^3+^ and Y_2_O_3_/Y_2_O_2_S:Eu^3+^(YO/YOS-2) monitored at (**a**) 615 and (**b**) 630 nm.

Figure 6(a) shows the excitation spectra of Y_2_O_3_:Eu^3+^ and Y_2_O_3_/Y_2_O_2_S:Eu^3+^ monitored at 620 nm. Obviously, the excitation spectrum of Y_2_O_3_/Y_2_O_2_S:Eu^3+^ was different from that of Y_2_O_3_:Eu^3+^. The broad band centered at ~338 nm was due to the host lattice of Y_2_O_2_S. Figure 6(b) shows the excitation spectra of Y_2_O_3_:Eu^3+^ and Y_2_O_3_/Y_2_O_2_S:Eu^3+^ monitored at 630 nm. The excitation spectrum of Y_2_O_3_:Eu^3+^ was dominated by the excitation band centered at 259 nm, while the excitation spectrum of Y_2_O_2_S:Eu^3+^ was dominated by the excitation band centered at 338 nm.

**Figure 7:**
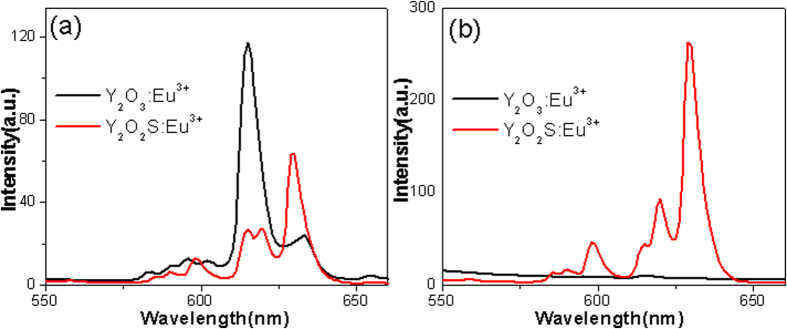
Emission spectra of Y_2_O_3_:Eu^3+^ and Y_2_O_3_/Y_2_O_2_S:Eu^3+^ (YO/YOS-2) excited at (**a**) 259 and (**b**) 338 nm.

Figure 7(a) shows the emission spectra of Y_2_O_3_:Eu^3+^ and Y_2_O_3_/Y_2_O_2_S:Eu^3+^ (YO/YOS-2) excited at 259 nm. For the Y_2_O_3_:Eu^3+^, the ^5^D_0_ → ^7^F_0_ (~583 nm), ^5^D_0_ → ^7^F_1_ (509–602 nm), and ^5^D_0_ → ^7^F_2_ (614–633 nm) transitions of the Eu^3+^ ions were observed. The luminescence was dominated by the emission at ~615 nm. The ^5^D_0_ → ^7^F_1_ emission was split into three sub-bands due to local fields around Eu^3+^ and their separations depend on the energy for the direct excitation from the ^7^F_0_ ground level to the ^5^D_0_ excited level. For the Y_2_O_3_/Y_2_O_2_S:Eu^3+^, the ^5^D_0_ → ^7^F_0_ and ^5^D_0_ → ^7^F_1_ showed a little red-shift. The luminescence was dominated by the emission at ~630 nm. In addition, the emission at ~615 nm was split into two sub-bands. Figure 7(b) shows the emission spectra of Y_2_O_3_:Eu^3+^ and Y_2_O_3_/‘’Y_2_O_2_S:Eu^3+^ (YO/YOS-2) excited at 338 nm. The luminescence intensity of Y_2_O_3_:Eu^3+^ has been enhanced by hybridization with Y_2_O_2_S:Eu^3+^.

**Figure 8:**
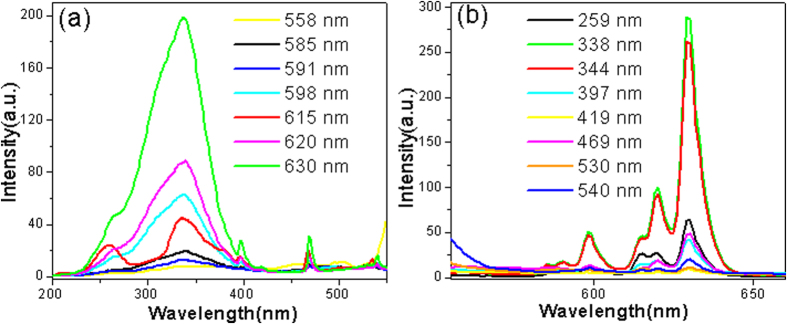
The (**a**) excitation and (**b**) emission spectra of the Y_2_O_3_/Y_2_O_2_S:Eu^3+^ (YO/YOS-2).

**Figure 9:**
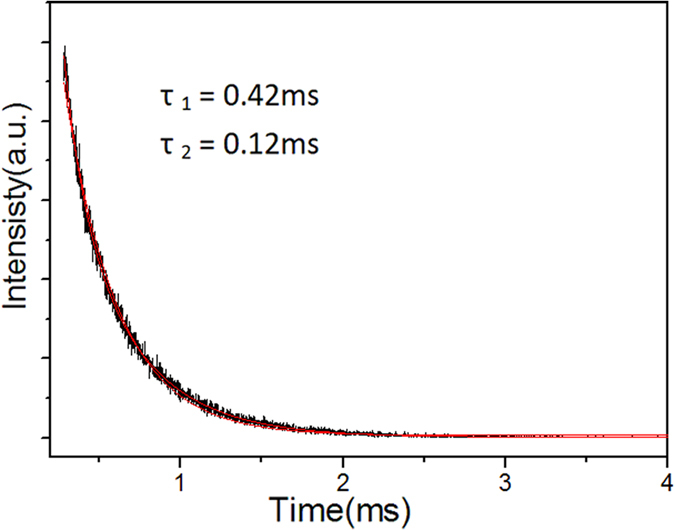
The luminescence decay curve for the Y_2_O_3_/Y_2_O_2_S:Eu^3+^ (YO/YOS-2) excited at 280 nm and monitored at 620 nm.

Figure 8(a) shows the excitation spectra of the Y_2_O_3_/Y_2_O_2_S:Eu^3+^ nanocomposites monitored at different wavelengths. It is noted that the excitation spectrum monitored at 615 nm was different from those monitored at other wavelengths. The results further prove that Y_2_O_3_:Eu^3+^ and Y_2_O_2_S:Eu^3+^ coexist in Y_2_O_3_/Y_2_O_2_S:Eu^3+^ nanocomposites. Figure 8(b) shows the emission spectra of the Y_2_O_3_/Y_2_O_2_S:Eu^3+^ nanocomposites excited at different wavelengths. When the excitation wavelength was 338 nm, the emission intensities were the strongest. Figure 9 shows the luminescence decay curve for the Y_2_O_3/_Y_2_O_2_S:Eu^3+^ nanocomposites excited at 280 nm and monitored at 620 nm. It is noted that the decay curve cannot be fitted with the single exponential function, while a biexponential function may reproduce the decay data well and lead to two lifetimes of 0.42 and 0.12 ms. The relative contribution of the exponentials to the decay of the hybrid spheres is about 0.51:0.49.

**Figure 10:**
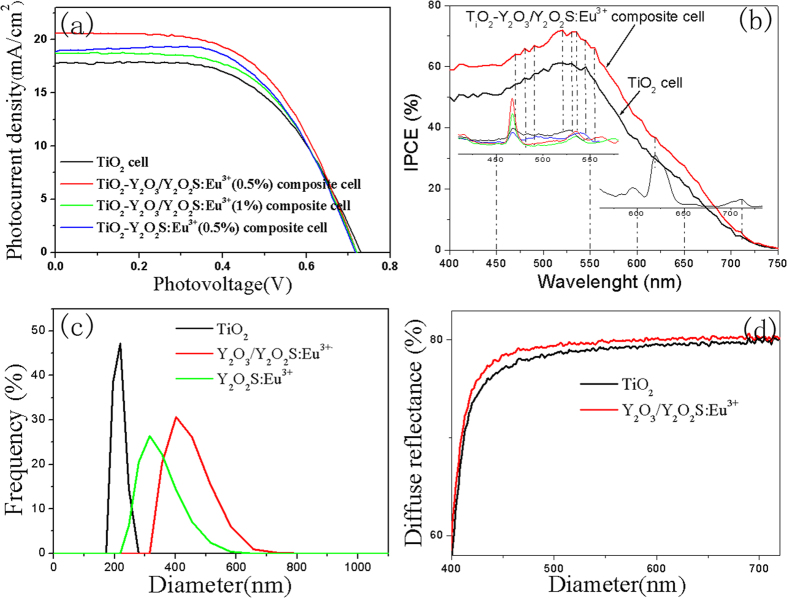
(**a**) The J-V curves and (**b**) IPCE spectra of pure TiO_2_ cell, TiO_2_-Y_2_O_3_/Y_2_O_2_S:Eu^3+^, and TiO_2_-Y_2_O_2_S:Eu^3+^ composite cells under simulated solar light radiation. (**c**) Dynamic light scattering (DLS) of Y_2_O_3_/Y_2_O_2_S:Eu^3+^ nanocomposites in water. (**d**) Comparison of diffuse reflectance spectra of TiO_2_ and TiO_2_-Y_2_O_3_/Y_2_O_2_S:Eu^3+^ photoanodes.

In order to investigate the effects of Y_2_O_3_/Y_2_O_2_S:Eu^3+^ on the photoelectric properties of DSSCs, the DSSC prototype devices were fabricated by using N719-sensitised TiO_2_-Y_2_O_3_/Y_2_O_2_S:Eu^3+^ composite electrodes. Figure 10(a) shows the photocurrent density-voltage (J-V) curves of pure TiO_2_ cell, TiO_2_-Y_2_O_3_/Y_2_O_2_S:Eu^3+^ composite cells, and TiO_2_-Y_2_O_2_S:Eu^3+^ composite cell. The corresponding values of the open-circuit voltage (V_oc_), short-circuit current density (J_sc_), fillfactor (FF), and overall conversion efficiency (η), obtained from the curves of solar cells, are shown in Table 2. The result indicated that the photoelectric conversion efficiencies of the TiO_2_-Y_2_O_3_/Y_2_O_2_S:Eu^3+^ composite cells were higher than those of pure TiO_2_ cell and TiO_2_-Y_2_O_2_S:Eu^3+^ composite cell. The best photoelectric conversion performance was observed when the mass concentration of Y_2_O_3_/Y_2_O_2_S:Eu^3+^ was 0.5%.

Presumably, three mechanisms might be responsible for the enhancement of the efficiencies of TiO_2_-Y_2_O_3_/Y_2_O_2_S:Eu^3+^ composite cells. (a) The improvement of the efficiencies of the TiO_2_-Y_2_O_3_/Y_2_O_2_S:Eu^3+^ composite cells were related to the luminescence of Y_2_O_3_/Y_2_O_2_S:Eu^3+^ nanocomposites. However, the results of the incident photon to current spectra (IPCE) indicated that the luminescence of Y_2_O_3_/Y_2_O_2_S:Eu^3+^ only has a little effect on the performance improvement, as shown in Fig. 10(b). (b) The enhancement of the efficiencies of the TiO_2_-Y_2_O_3_/Y_2_O_2_S:Eu^3+^ composite cells were related to the light scattering of Y_2_O_3_/Y_2_O_2_S:Eu^3+^, as shown in Fig. 10(c,d). (c) It is noted that the sintering process was necessary during preparation of the photoelectrode, which has been described in the Experimental section. And thus, some Ti^4+^ ions will be substituted by S^6+^ during the sintering process, which was beneficial for enhancing photoelectric properties^31^. In addition, the decrease of the efficiency of TiO_2_-1%Y_2_O_3_/Y_2_O_2_S:Eu^3+^ was related to the decrease of the amount of dye adsorption and lower interfacial electron transfer^32^.

In summary, Y_2_O_3_:Eu^3+^ nanocrystals were synthesized by a hydrothermal method first, and then Y_2_O_2_S:Eu^3+^ nanocrystals and Y_2_O_3_/Y_2_O_2_S:Eu^3+^ nanocomposites were obtained by reducing Y_2_O_3_:Eu^3+^ nanocrystals. The luminescence of Y_2_O_3_/Y_2_O_2_S:Eu^3+^ excited at 338 nm was much stronger than that of Y_2_O_3_:Eu^3+^ nanocrystals. Compared with Y_2_O_3_:Eu^3+^, the ^5^D_0_ → ^7^F_0_ and ^5^D_0_ → ^7^F_1_ emissions of Y_2_O_3_/Y_2_O_2_S:Eu^3+^ showed a little red-shift. In addition, the emission at ~615 nm of Y_2_O_3_/Y_2_O_2_S:Eu^3+^ was split into two sub-bands. In addition to the aforementioned luminescence properties, these Y_2_O_3_/Y_2_O_2_S:Eu^3+^ nanocomposites also can be chosen to design TiO_2_-Y_2_O_3_/Y_2_O_2_S:Eu^3+^ composite cell, which have the ability to improve the photoelectric conversion efficiency. We suggested that the enhancement of the efficiency of the TiO_2_-Y_2_O_3_/Y_2_O_2_S:Eu^3+^ composite cell was mainly related to the light scattering of Y_2_O_3_/Y_2_O_2_S:Eu^3+^ nanocomposites.

## Methods

### Preparation of samples

All of the chemicals used in this paper were analytical grade and used as received without further purification. In the synthesis of Y_2_O_3_/Y_2_O_2_S:Eu^3+^, 3 mL of Ln(NO_3_)_3_ (Ln = Y and Eu) aqueous solution (0.5 mol/L) was added to 3 mL deionized water, and the solution was thoroughly stirred, then an aqueous solution of NaOH (0.25 M) was added into the above solution. Subsequently, the milky colloidal solution was transferred to a 50 mL Teflon-lined autoclave, and heated at 100 °C for 5 h. The systems were then allowed to fast cool to room temperature. The final products were collected by means of centrifugation, washed with deionized water and ethanol, dried at 80 °C for 4 h in air. And then the Y_2_O_3_:Eu^3+^ precursor was obtained. 0.1 g of the Y_2_O_3_:Eu^3+^ precursor and some (1.0, 1.5, and 2.0 g) sulfur powder were put into a porcelain boat with different proportions of sulfur powder, and then sintered at 600 °C for 1 h in N_2_ atmosphere.

### Fabrication of photoelectrodes

Fabrication of photoelectrode and the assembly of DSSCs: several pastes, from homogeneously mixing Y_2_O_3_/Y_2_O_2_S:Eu^3+^ and TiO_2_ (Degussa P25) into 1.5 mL of TiO_2_ colloid. The TiO_2_ colloid was prepared following the previously published synthesis procedure^33^. A screen-printed double layer of TiO_2_-Y_2_O_3_/Y_2_O_2_S:Eu^3+^ was used as the photoanode. The first layer of TiO_2_-Y_2_O_3_/Y_2_O_2_S:Eu^3+^ was prepared by a doctor-blade method on the FTO substrate and then sintered at 450 °C for 30 min. Subsequently, the second layer of TiO_2_-Y_2_O_3_/Y_2_O_2_S:Eu^3+^ was covered on the first TiO_2_-Y_2_O_3_/Y_2_O_2_S:Eu^3+^ film and then sintered at 450 °C for 30 min again. The sensitization of the photoelectrodes was achieved by immersing them into 0.5 mM ((C_4_H_9_)_4_N)_2_[Ru(4-carboxy-4′-carboxylate-2,2′ bipyridine)_2_ (NCS)_2_] dye (N719, Solaronix SA, Switzerland) in acetonitrile and tert-butanol (volume ratio, 1:1) for 48 h at room temperature. The Pt counter electrodes were prepared following the previous literature^34^. The dye-sensitized photoanode was assembled with a Pt counter electrode into a sandwichtype cell. The sandwich-type cell was further fixed together with epoxy resin. The space between the electrodes was filled with the electrolyte, which comprised 0.6 M 1-propyl-2,3-dimethyl-imidazolium iodide, 0.05 M I_2_, 0.1 M LiI, and 0.5 M tert-butylpyridine (TBP) in 3-methoxypropionitrile (3-MPN), by capillary action.

### Materials Characterization

The crystal structure was analyzed by a Rigaku (Japan) D/MAX-rA X-ray diffraction meter equipped with graphite monochromatized Cu Kα radiation (λ = 1.541874 Å), keeping the operating voltage and current at 40 kV and 40 mA, respectively. The sizes and morphologies of the final products were determined by using a JEOL JEM-2010F transmission electron microscope (TEM) operated at 200 kV. X-ray photoelectron spectroscopy (XPS) analysis was performed using a VG ESCALABMK II with a Mg KR (1253.6 eV) achromatic X-ray source. The photoluminescence spectra were recorded using a Hitachi F-4600 fluorescence spectrophotometer at room temperature. For comparison of the luminescence properties of different samples, the luminescence spectra were measured with the same instrument parameters (2.5 nm for slit width and 700 V for PMT voltage). The luminescence decay curve was recorded by a Spex 1403 spectrometer under the excitation of a third harmonic (355 nm) of a Nd:YAG pulsed laser.

### Photovoltaic properties

Photovoltaic measurements were carried out with a solar simulator (Oriel, USA) equipped with an AM 1.5 G radiation (1 sun conditions, 100 mW cm^−2^) filter was used as the light source. The irradiation area of DSSCs is 0.09 cm^2^. The electron transport and recombination properties were measured by intensity-modulated photocurrent spectroscopy (IMPS) and intensity-modulated photovoltage spectroscopy (IMVS) (Zahner Elektrik, Germany).

## Additional Information

**How to cite this article**: Yuan, G. *et al*. *In situ* synthesis, enhanced luminescence and application in dye sensitized solar cells of Y_2_O_3_/Y_2_O_2_S:Eu^3+^ nanocomposites by reduction of Y_2_O_3_:Eu^3+^. *Sci. Rep.* **6**, 37133; doi: 10.1038/srep37133 (2016).

**Publisher’s note:** Springer Nature remains neutral with regard to jurisdictional claims in published maps and institutional affiliations.

## Figures and Tables

**Table 1:** The effect of sulfur powder contents on the products.

Samples	Y_2_O_3_:Eu^3+^ precursor	Sulfur powder	Products
YO	0.1 g	0 g	Y_2_O_3_:Eu^3+^
YO/YOS-1	0.1 g	1.0 g	(Y_2_O_3_+Y_2_O_2_S):Eu^3+^
YO/YOS-2	0.1 g	1.5 g	(Y_2_O_3_+Y_2_O_2_S):Eu^3+^
YOS	0.1 g	2.0 g	Y_2_O_2_S:Eu^3+^

**Table 2:** Solar cell parameters of TiO_2_, TiO_2_-Y_2_O_3_/Y_2_O_2_S:Eu^3+^, and TiO_2_-Y_2_O_2_S:Eu^3+^ cells under simulated solar light radiation.

DSSCs	*J*_SC_(mA cm^−2^)	*V*_OC_(V)	FF	*η*(%)
TiO_2_	17.79	0.73	0. 567	7.37
TiO_2_-(0.5%)Y_2_O_3_/Y_2_O_2_S:Eu^3+^	20.60	0.72	0. 565	8.38
TiO_2_-(1%)Y_2_O_3_/Y_2_O_2_S:Eu^3+^	18.73	0.72	0. 573	7.72
TiO_2_-(0.5%)Y_2_O_2_S:Eu^3+^	19.00	0.72	0.579	7.92
